# Increased expression of CXCL10 and CCL3 salivary gland chemokines in primary Sjögren’s syndrome detected and systematically quantified using RNAscope^®^ *in situ* hybridization

**DOI:** 10.1093/cei/uxae087

**Published:** 2024-10-22

**Authors:** Hanne Borge, Ingrid Beate Ringstad, Lara A Aqrawi, Siren Fromreide, Harsh Nitin Dongre, Hilde Kanli Galtung, Janicke Liaaen Jensen, Kathrine Skarstein

**Affiliations:** Gade Laboratory for Pathology, Department of Clinical Medicine, University of Bergen, Bergen, Norway; Faculty of Dentistry, Department of Oral Surgery and Oral Medicine, University of Oslo, Oslo, Norway; Department of Health Sciences, Kristiania University College, Oslo, Norway; Gade Laboratory for Pathology, Department of Clinical Medicine, University of Bergen, Bergen, Norway; Gade Laboratory for Pathology, Department of Clinical Medicine, University of Bergen, Bergen, Norway; Faculty of Dentistry, Institute of Oral Biology, University of Oslo, Oslo, Norway; Faculty of Dentistry, Department of Oral Surgery and Oral Medicine, University of Oslo, Oslo, Norway; Gade Laboratory for Pathology, Department of Clinical Medicine, University of Bergen, Bergen, Norway; Department of Pathology, Haukeland University Hospital, Bergen, Norway

**Keywords:** Sjögren’s syndrome, chemokines, CXCL10, CCL3, RNAscope®, *in situ* hybridization, RT-qPCR, immunohistochemistry, salivary gland biopsies, saliva

## Abstract

Primary Sjögren’s syndrome is a chronic inflammatory disease characterized by the destruction of exocrine glands. We have previously shown significantly upregulated levels of CXCL10 and CCL3 chemokines in saliva from Sjögren’s syndrome patients. In this study, we examined the expression pattern and localization of these chemokines at the site of inflammation in patients’ minor salivary glands using novel RNAscope® *in situ* hybridization. Minor salivary glands from 33 primary Sjögren’s syndrome patients and 22 non-Sjögren’s syndrome (non-SS) sicca controls were included. The biopsies were formalin-fixed, paraffin-embedded, and histopathologically evaluated. The CXCL10 and CCL3 mRNA expression in the glandular tissue was investigated using reverse transcription quantitative real-time polymerase chain reaction followed by an RNAscope® *in situ* hybridization. The mRNA expression of CXCL10 was higher than CCL3 in all patients. Significantly elevated expression of CXCL10 and CCL3 was detected in patients that also expressed autoantibody positivity and a positive biopsy for mononuclear cell infiltrates when compared with non-SS sicca controls. CXCL10 was localized as clusters within focal infiltrates as well as adjacent to acinar and ductal epithelium, while CCL3 was expressed as scattered single mRNA molecules in focal infiltrates and in acinar cells. Our findings suggest CXCL10 as a possible disease biomarker in primary Sjögren’s syndrome due to its upregulated expression in both saliva and minor salivary glands of patients and the localization in the tissue. This should be re-assessed in a larger primary Sjögren’s syndrome patient cohort, followed by additional functional studies to further validate its potential as a disease biomarker.

## Teaser

In this study, we found an upregulation of CXCL10 and CCL3 in Primary Sjögren’s syndrome (pSS) patients compared with non-SS sicca controls. Additionally, our results implicate that there is a correspondence between the expression of CXCL10 and the size of the inflammatory infiltrate in the pSS cohort. CXCL10 expression also correlates with more severe clinical manifestations and might be a predictor of glandular destruction in pSS and a potential disease biomarker.

## Graphical Abstract

**Graphical Abstract:**
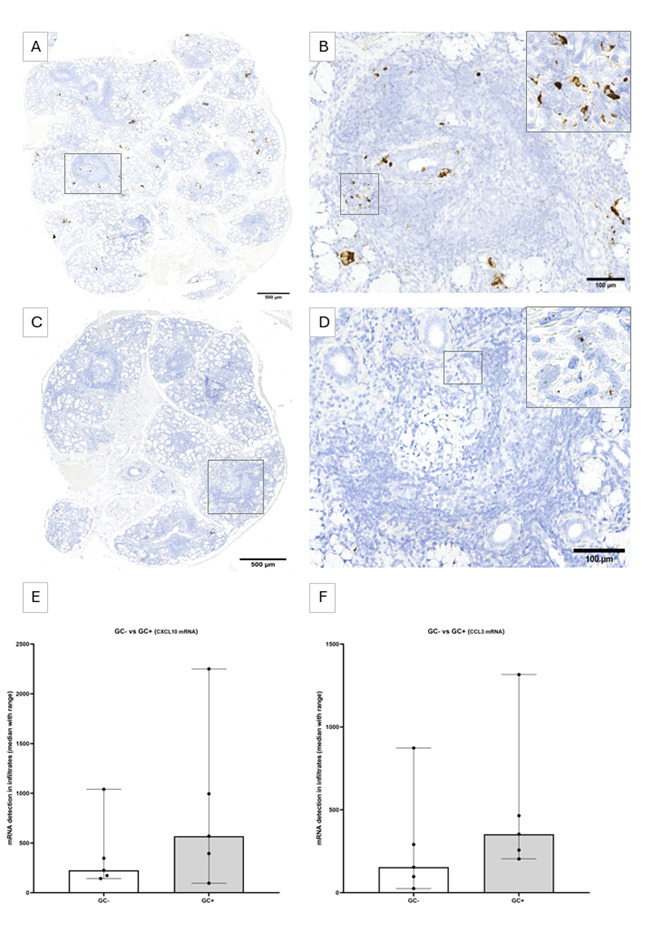

## Background

Primary Sjögren’s syndrome (pSS) is a rheumatic autoimmune disease affecting several tissues and organs where the salivary and lacrimal glands are the first site of inflammation, causing the common symptoms of dry eyes and dry mouth [1]. A characteristic feature of the disease is the lymphocytic infiltration in patients’ exocrine glands that leads to the accumulation of mononuclear cells, resulting in the consequent destruction of the glandular tissue. This infiltration can affect both the major and minor salivary glands (MSG). Histological evaluation of the MSG is, therefore, an important part of pSS diagnosis [2–4]. These glandular infiltrates may comprise T cells, B cells, plasma cells [5, 6], and antigen-presenting cells, such as macrophages and dendritic cells [6, 7].

More information can be generated on the pathological features of pSS by investigating the chemokine expression of the salivary gland tissue, both within the inflammatory infiltrates and the ductal and acinar epithelium surrounding the lymphocytes. Interestingly, chemokines are one of the key mediators in the pathological processes associated with autoimmunity [8, 9]. The structure of the different chemokines categorises them into different subfamilies, including the CXC- and CC-family. These are the two main chemokine subfamilies involved in the pSS disease pattern. Motif chemokine ligand 10 (CXCL10/IP-10) is one of the central CXC chemokines induced by IFN-γ signalling pathway that binds to the CXCR3 receptor highly expressed on activated T helper cell type 1 (Th1) inflammatory cells, thereby promoting a Th1 inflammatory response [10]. This CXCR3 receptor is expressed on both CD4 and CD8 positive T cells, where CXCL10 binds to this receptor to promote the proliferation of T cells from lymphoid organs to the site of inflammation [11, 12]. The T lymphocytes are predominant in the periductal infiltration of the salivary glands, and CXCL10 may therefore be a central chemokine involved in the initial development of the mononuclear cell infiltrates destroying the glandular structures of pSS patients [10, 13]. On the other hand, motif chemokine ligand 3 (CCL3/MIP-1α) from the CC subfamily of chemokines reacts through the CCR1, CCR5, and CCR9 receptor on activated and memory T cells [14]. It has been documented that CCL3 also participates in the regulation of inflammatory diseases [14]. CCL3 has both chemotactic and inflammatory potential and is mainly produced by macrophages, lymphocytes, and other cell types involved in mediation and regulation of inflammation [15].

We have previously demonstrated a significant upregulation of CXCL10 and CCL3 in the saliva of pSS patients using multiplex immunobeads assay technology [2]. By exploring the salivary gland tissue in this study for expression of these two specific chemokines, in an already well-established and characterized cohort, we aim to gain more information on the histological features and pathological implications within MSG tissue from both pSS patients and non-Sjögren’s sicca controls (non-SS). These chemokines have been difficult to detect through immunohistochemistry, due to unsatisfying sensitivity and specificity of the antibodies currently available. Hence, we have now used RNAscope® *in situ* hybridization for the first time in pSS to systematically quantify the level of CXCL10 and CCL3 mRNA expression and localization in glandular pSS MSG tissue [16, 17]. By doing so, we can establish a deeper understanding of the relationship between the salivary gland microenvironment and how this correlates with the expression of both CXCL10 and CCL3 at the site of inflammation in pSS. This may also aid in introducing a potential biomarker for the disease that can be detected in patients’ saliva, as there is an unmet need for a more non-invasive diagnostic element in pSS assessment today.

## Materials and methods

### Study population

MSG samples from 20 pSS patients and 12 non-SS sicca controls were included in the reverse transcription quantitative real-time polymerase chain reaction (RT-qPCR) analysis. Additionally, MSG biopsies from 13 pSS patients and 10 non-SS sicca controls were included in the RNAscope® cohort. All pSS patients fulfilled the American College of Rheumatology and the European League against Rheumatism criteria. As part of the diagnostic procedure, patients were recruited at the Department of Oral Surgery and Oral Medicine, University of Oslo, between years 2011 and 2022.

Lower labial MSG biopsies were harvested by an oral surgeon (J.L.J.) at the University of Oslo. The biopsies were formalin-fixed and sent to the Gade Laboratory of Pathology, Haukeland University Hospital, Bergen, where they were paraffin embedded prior to haematoxylin and eosin staining. Diagnostic analyses of the tissue sections were then performed by an oral pathologist (K.S.) at the University of Bergen. The sections were examined for focal mononuclear cell infiltrates, atrophy/fibrosis, fat infiltration, and germinal centre (GC)-like structures. A focus score was set for each patient based on the number of mononuclear cell infiltrates with ≥50 mononuclear cells per 4 mm^2^ glandular tissue area. Fat infiltration was scored and analysed to evaluate the level of adipose tissue replacement within the normal MSG tissue, as described below. The non-SS subjects displayed little to no pathological changes in their MSG tissue, showing in general only minor structural alterations that did not dominate the features of the glands. The level of fatty infiltration (FI), fibrosis, or atrophy was more evident in pSS patients as compared with non-SS sicca controls. Moreover, both study groups were age and sex matched.

Supplemental immunohistochemical analysis was carried out when suspecting GC-like structures that appeared within the mononuclear infiltrates, using CD21 antibody for the identification of follicular dendritic cells.

Clinical characteristics were obtained from medical examinations and patients’ charts at the Department of Oral Surgery and Oral Medicine, University of Oslo, and Martina Hansens Hospital. Medical data were obtained from the referring physician. This included information regarding the presence of anti-Ro/SSA and anti-La/SSB autoantibodies, levels of saliva and tear secretion, as well as subjective sensation of dryness in mouth and eyes. These clinical and laboratorial characteristics are presented in Table 1 for pSS patients and Table 2 for non-SS sicca controls. All participants have given their informed consent according to the ethical declarations for participation in this study. Moreover, the study has been approved by the Regional Medical Ethical Committee (2010/1292 and 2015/363).

**Table 1:** characteristics of the pSS patients included in the study

Patient no.	Age (years) ^a^	Sex	ANA ^b^	Anti-SSA ^b^	Anti-SSB ^b^	Focus score ^c^	GC	FI-score ^d^	Schirmer’s test ^e^	Saliva secretion ^f^	Dry mouth ^g^	Dry eyes ^g^
1	54	F	−	+	+	12	+	−	+	+	+	+
2	43	M	+	+	−	0	−	−	+	+	+	+
3	46	F	+	+	+	1	−	−	+	+	+	+
4	49	F	+	+	−	1	−	−	−	+	−	+
5	65	F	+	+	−	1	+	−	+	−	−	+
6	60	F	+	+	−	1	−	−	+	+	−	+
7	30	M	+	+	−	1	−	−	+	+	+	+
8	74	F	+	+	−	3	+	−	+	−	+	+
9	39	F	−	−	−	2	+	−	+	+	+	+
10	35	F	+	+	−	3	+	−	+	+	−	−
11	80	M	+	+	+	1	+	−	+	+	−	+
12	26	F	+	+	−	1	−	−	−	+	+	−
13	52	F	+	+	−	3	+	−	+	+	−	+
14	55	F	−	−	−	1	−	−	+	−	+	+
15	49	F	+	+	−	0	−	−	−	+	+	+
16	61	F	−	−	−	1	−	−	+	−	+	+
17	63	F	+	+	−	1	−	−	+	+	+	+
18	50	F	−	−	−	1	−	−	+	+	+	+
19	33	M	+	+	−	0	−	−	−	+	+	+
20	33	M	+	+	−	0	−	−	−	+	+	+
21	50	F	+	+	+	8	+	1	+	−	−	−
22	41	F	+	+	+	2	−	1	+	+	+	+
23	23	F	+	+	+	1	−	0	−	+	+	−
24	49	F	+	+	+	2	+	1	−	−	−	−
25	43	F	+	+	+	10	+	1	+	+	+	+
26	46	F	+	+	+	2	+	2	+	−	+	+
27	72	F	+	+	−	1	−	1	+	−	−	+
28	55	F	+	+	−	1	−	2	+	+	+	+
29	45	F	+	−	−	2	+	1	+	+	+	+
30	70	F	+	−	−	1	−	1	+	+	+	+
31	54	F	+	+	−	0	−	1	+	+	+	+
32	64	F	+	+	−	0	−	1	+	+	+	+
33	35	F	+	+	−	3	−	0	+	+	−	−

GC: germinal centre-like structures.

^a^Age: age at biopsy.

^b^Autoantibody levels were measured in patient serum using ELISA.

^c^Score represents the number of focal infiltrates/4 mm^2^ glandular tissue area with ≥50 mononuclear cells.

^d^The level of FI in the salivary gland tissue, 0 = low, 1 = moderate, and 2 = prominent.

^e^Normal tear flow > 5 mm/5 min. ‘+’ indicates eye dryness and tear secretion ≤5 mm/5 min.

^f^Normal salivary flow > 1.5 mL/15 min. ‘+’ indicates dryness and level of unstimulated whole salivary secretion ≤1.5 mL/15 min.

^g^Patient reported symptoms of dryness.

**Table 2:** characteristics of non-SS sicca control subjects

Subject no.	Age (years) ^a^	Sex	ANA ^b^	Anti-SSA ^b^	Anti-SSB ^b^	Focus score ^c^	GC	FI-score ^d^	Schirmer’s test ^e^	Saliva secretion ^f^	Dry mouth ^g^	Dry eyes ^g^
1	75	F	−	−	−	0	−	−	+	+	+	+
2	56	F	−	−	−	0	−	−	−	+	+	+
3	61	F	−	−	−	0	−	−	+	+	+	+
4	41	F	−	−	−	0	−	1	+	+	+	+
5	42	F	−	−	−	0	−	−	−	+	+	+
6	50	F	−	−	−	0	−	−	+	+	+	+
7	61	F	−	−	−	0	−	−	+	+	+	+
8	48	F	+	−	−	0	−	−	+	−	+	+
9	39	F	−	−	−	0	−	−	+	+	−	−
10	32	F	+	−	−	<1	−	−	−	+	+	+
11	45	M	−	−	−	<1	−	−	+	−	+	+
12	51	F	−	−	−	0	−	−	+	+	+	+
13	54	F	−	−	−	<1	−	−	+	+	+	+
14	65	F	−	−	−	0	−	2	−	−	+	+
15	39	F	−	−	−	0	−	1	+	+	+	+
16	44	F	−	−	−	0	−	1	−	+	+	+
17	56	F	−	−	−	0	−	2	+	+	+	+
18	58	F	−	−	−	0	−	2	+	+	+	+
19	38	F	−	−	−	0	−	0	−	+	+	+
20	53	F	−	−	−	0	−	2	+	+	+	+
21	67	F	−	−	−	0	−	0	+	+	+	+
22	51	F	−	−	−	0	−	0	+	−	+	+
23	50	F	+	−	−	0	−	1	+	+	+	+

GC: germinal centre-like structures.

^a^Age: age at biopsy.

^b^Autoantibody levels were measured in patient serum using ELISA.

^c^Score represents the number of focal infiltrates/4 mm^2^ glandular tissue area with ≥50 mononuclear cells.

^d^The level of FI in the salivary gland tissue was scored, 0 = low, 1 = moderate, and 2 = prominent.

^e^Normal tear flow > 5 mm/5 min. ‘+’ indicates eye dryness and tear secretion ≤ 5 mm/5 min.

^f^Normal salivary flow > 1.5 mL/15 min. ‘+’ indicates dryness and level of unstimulated whole salivary secretion ≤1.5 mL/15 min.

^g^Patient reported symptoms of dryness.

### Detection of mRNA expression levels in MSG using RT-qPCR

#### Tissue preparation and RNA isolation

RNA isolation and RT-qPCR were performed on MSG of 20 pSS patients and 13 non-SS sicca controls. The salivary glands were extracted from the patients’ lower lip and put directly into collection tubes containing RNAlater (Sigma-Aldrich, St. Louise, MO, USA). The tubes were stored at 4°C before being frozen at −150°C. The glandular tissue from each participant was homogenized using lysis buffer (Qiagen, Valencia, CA, USA) and 2-mercaptoethanol (Sigma-Aldrich, Darmstadt, Germany) before stored at −80°C. Extraction of RNA was done using RNeasy Mini kit (Qiagen) following the manufacturers’ instructions. RNA purity and quantification were measured using a NanoDrop spectrophotometer (NanoDrop Technologies Inc., Wilmington, DE, USA).

#### Quantitative real-time polymerase chain reaction

A total of 200 ng RNA was used per 10 µl reverse transcriptase to make cDNA. The synthesis of cDNA was generated using Reverse Transcription Core Kit (Eurogentec, Seraing, Belgium). A mix of 10× buffer, MgCl_2_, dNTP, random nonamer, RNase inhibitor, and EuroScript reverse transcriptase enzyme was made and distributed in all tubes before adding 200 ng RNA and RNase-free water. A total volume of 20 µl was used for each cDNA synthesis, starting with 10 min at 25°C, then 30 min at 48°C, and terminated by incubation for 5 min at 95°C.

The mRNA levels of CXCL10, CCL3, and GAPDH were detected using Assay-on Demand TaqMan Gene Expression Assay (AOD, Applied Biosystems, Foster City, CA). The RT-qPCR reactions were performed using a 96-well PCR-reaction plate on AriaMX Real-Time PCR System (Agilent Technologies, Santa Clara, CA) with 40 cycles (95°C for 15 s and 60°C for 1 min) after an initial 10 min incubation of 95°C.

Each RT-qPCR reaction consisted of 1× AOD mix, 1× qPCR Takyon Low Rox Probe MasterMix dTTP Blue (Eurogentec, Seraing, Belgium), and 10 µl cDNA (diluted 1:2.5 with H_2_O) as the template. The data were exported to an Excel-sheet, and results were normalized to GAPDH as an endogenous control. Duplicates were run for every individual sample. The average amount of the target gene was calculated for each duplicate. This value was then standardized to the mRNA-value of GAPDH using the equation ΔCq=[Cq(target gene) − Cq(reference gene)] and presented as $2^{\left(-\Delta Cq\right)}$ .

#### RNAscope® staining method and analysis

*In situ* hybridization was performed on 13 pSS patients and 10 non-SS sicca controls. The protocol used was according to the RNAscope® 2.5 HD Brown Detection kit [Advanced Cell Diagnostics (ACD), Hayward, CA, USA], and 4 μm sections of formalin-fixed, paraffin-embedded MSGs were sectioned on a microtome (Leica RM2235) before mounted on Superfrost Plus slides (Thermo Scientific, Waltham, MA, USA). Furthermore, the sections were baked at 60°C for 1h in an air oven and deparaffinised using xylene in 2 × 5 min followed by 2 × 1 min 100% alcohol. A hydrophobic barrier was created using the ImmEdge™ pen (Vector Laboratories, Newark, CA, USA) after airdrying the slides for 5 min at room temperature.

Next, the specimens were pre-treated with hydrogen peroxide for 10 min at room temperature. Target retrieval was performed using the RNAscope® Target Retrieval Reagent for 15 min at 98–102°C. This was followed by RNAscope® Protease Plus treatment for 30 min at 40°C in the HybEZ™ oven before incubation with the target probe against CXCL10 mRNA (ACD) or CCL3 mRNA (ACD) for 2 h in the HybEZ™ oven at 40°C. The signal was then amplified, and the results were visualized using 3,3’-diaminobenzidine (DAB). After signal detection, the sections were counterstained with haematoxylin (Agilent Dako, Santa Clara CA, USA), dehydrated through 100% alcohol and xylene, and mounted using Pertex (Histolab, Askim, Sweden). A probe for the housekeeping gene Peptidylprolyl Isomerase was used as positive control for the mRNA quality, and DapB, a transcript from *Bacillus subtilis*, was used as a negative control probe. Positive staining was indicated by brown punctate dots in the cytoplasm or nucleus of the cell.

#### Dual staining method

To simultaneously visualize the CXCL10 mRNA together with CD3 positive T cells and CD68 positive macrophages, respectively, a combined approach of *in situ* hybridization and immunohistochemistry was performed. The RNAscope® RED assay in combination with the RNA-Protein co-detection ancillary kit (ACD) were used. The sections were deparaffinized and rehydrated as in the standard RNAscope® workflow (described above), and target retrieval was performed using RNAscope® co-detection target retrieval reagent for 15 min at 98–102°C. The sections were further treated with RNAscope® Protease Plus treatment for 30 min at 40°C in the HybEZ™ oven followed by incubation with CXCL10 mRNA (ACD), for 2 h at 40°C in the HybEZ™ oven. The standard RNAscope® workflow for signal amplification (as described above) was conducted, and signal detection was performed using fast red chromogen for visualization. This was followed by primary antibody incubation with either 1:100 anti-CD3 mouse clone F7.2.38 (Agilent Dako) or 1:20 anti-CD68 clone PG-M1 (Agilent Dako), in RNAscope® co-detection antibody diluent over night at 4°C. Next, the sections were incubated with Envision FLEX mouse linker (Agilent Dako), for 20 min, followed by Envision FLEX HRP secondary antibody (Agilent Dako), for 20 min at room temperature. Detection of signal was performed using DAB chromogen. The sections were further counterstained with haematoxylin (Agilent Dako), air dried for 15 min at 60°C, and treated with xylene before mounted with Pertex (Histolab).

#### Quantification of visualized mRNA transcripts using QuPath Software

The sections were scanned by a whole slide scanner (Hamamatsu NanoZoomer-XR, Hamamatsu City, Japan) and digital quantification was performed using QuPath software 0.3.2 [18]. The region of interest (ROI) was determined using Fischer et al. guidelines for annotating the inflammatory infiltrates within the glandular area before proceeding further with the analysis [3]. The annotation of ROIs was selected based on the histopathological evaluation of the mononuclear cell infiltrates with surrounding normal salivary gland tissue. One ROI represented one infiltrate, and the number of ROIs varied between 1 and 13 per patient and individually between CXCL10 and CCL3 staining due to the difference in MSG sections.

Staining vectors were estimated using the ‘Estimate staining vectors’ tool in QuPath software. A small rectangle was drawn in a representative area of the salivary gland to detect the correct and most accurate DAB staining vectors from the pixels in the selected area (Supplementary Fig. S1). The same process was done for estimating haematoxylin and background vectors. All the staining vectors were performed using the same scanned section and verified on three different sections.

After detecting the correct DAB, haematoxylin, and background staining vectors, an automatic cell detection script was made to standardize the mRNA detection throughout our samples. The automatic cell detection tool was used to segment cells in the tissue. A DAB detection classifier was created by adding the precise staining vectors to identify DAB spots and differentiate from pixels representing haematoxylin and background. Furthermore, a script was made to include both the cell detection and DAB detection classifier.

A number of mRNA spots identified was counted and classified according to the number of spots detected in each cell. If the cells were too clustered and/or had a very high mRNA expression level above 15+ mRNA spots, the script would mark the positive mRNA transcripts as subcellular clusters, in line with the manufacturer’s note on manual counting. The size of the cluster was divided by an ‘expected spot size’ to give an estimated number of spots. The cells were visually coloured yellow, blue, or black depending on the number of mRNA spots or clusters detected in the cells, while negative cells were detected with purple colour (Supplementary Fig. S2).

#### Manual quantification of the histopathological features and establishment of an index assessment of the salivary gland epithelium

Since a possible pathological feature in pSS is infiltration of adipose tissue in MSG, the fat infiltration in the MSG biopsies was analysed manually in QuPath software. An FI-score was set depending on the level of adipose tissue deposit in the gland. Here, 0 demonstrated little to negative fatty tissue, 1 exhibited moderate levels, while 2 showed prominent fat infiltration in the gland, as described in Aqrawi et al. [19].

The acinar epithelium was evaluated and scored with an index based on the level of mRNA expression in the acini of each biopsy and analysed for mRNA clusters regarding both CXCL10 and CCL3. Here, 0 was set as negative, 1 displayed low mRNA expression, 2 showed moderate, while 3 presented prominent levels of mRNA clusters of the specific chemokine.

#### Statistical analysis

Statistical analyses were performed using STATA software version 17.1 (College Station, TX, USA), and graphs were generated using GraphPad Prism 9.5.1 (GraphPad Software, San Diego, CA, USA). The Shapiro–Wilk test for normality was used to test the data for normal distribution. The data were considered not normally distributed with *P* < 0.05. The Mann–Whitney U test for nonparametric data was used to analyse the data for statistical significance between two groups. *P*-value ≤ 0.05 was set as significant and presented in graphs as *, while a *P*-value < 0.01 was visualized in the graphs with **.

## Results

### RT-qPCR analysis reveals significantly enhanced levels of CXCL10 and CCL3 mRNA in the MSG of pSS patients compared with non-SS sicca controls

**Figure 1:**
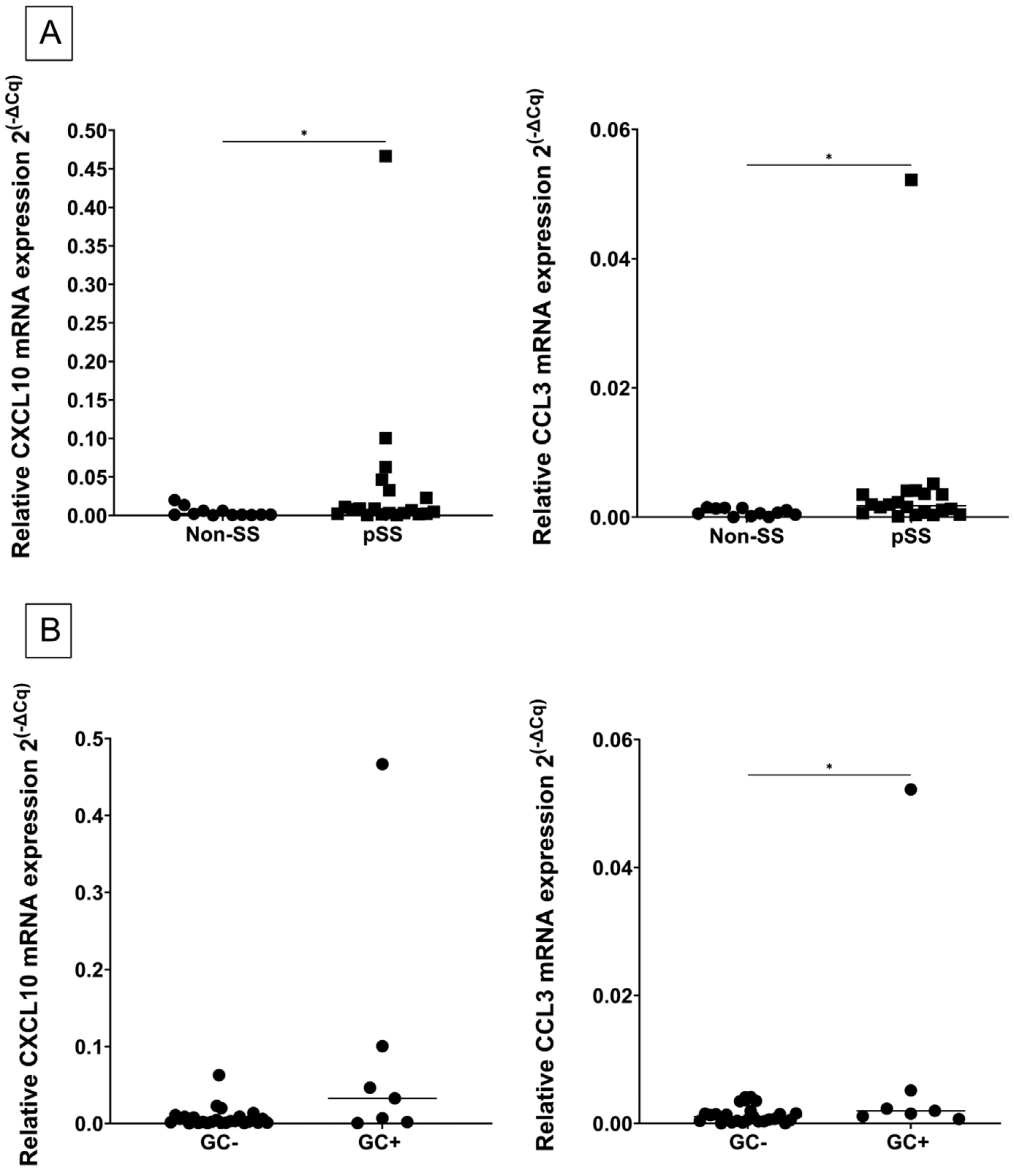
enhanced CXCL10 and CCL3 mRNA expression in salivary glands from pSS patients using RT-qPCR. (A) Significantly higher expression of CXCL10 and CCL3 mRNA in the MSG of pSS patients as compared with the non-SS sicca controls. Results are shown as median 2^(-∆Cq)^ values in the following order: non-SS sicca controls (circle), pSS (square). CXCL10 (0.00094; 0.0074), CCL3 (0.0007; 0.0017). (B) Higher expression of CXCL10 and significantly elevated CCL3 mRNA (*P* = 0.048) in MSG of GC-positive pSS patients as compared with GC-negative individuals. Results are shown as median 2^(-∆Cq)^ values in the following order: GC negative (GC−), GC positive (GC+). CXCL10 (0.003; 0.033), CCL3 (0.001; 0.002). Intergroup difference is significant at *P* < 0.05 and visualized as *

The mRNA expression level in MSG tissue was analysed through the application of RT-qPCR. Median comparison between pSS and non-SS sicca controls showed a significant upregulation of both CXCL10 and CCL3 mRNA levels in the MSG of pSS patients when compared with the non-SS sicca controls (Fig. 1A). Nonetheless, the highest difference between pSS and non-SS sicca subjects was seen when targeting CXCL10 mRNA. Notably, higher levels of CXCL10 and CCL3 mRNA expression were observed in pSS patients showing GC-like formations as compared with GC-negative individuals (Fig. 1B).

### Detection of CXCL10 and CCL3 mRNA transcripts within mononuclear cell infiltrates and GC-like formations in pSS using novel RNAscope® *in situ* hybridization

**Figure 2:**
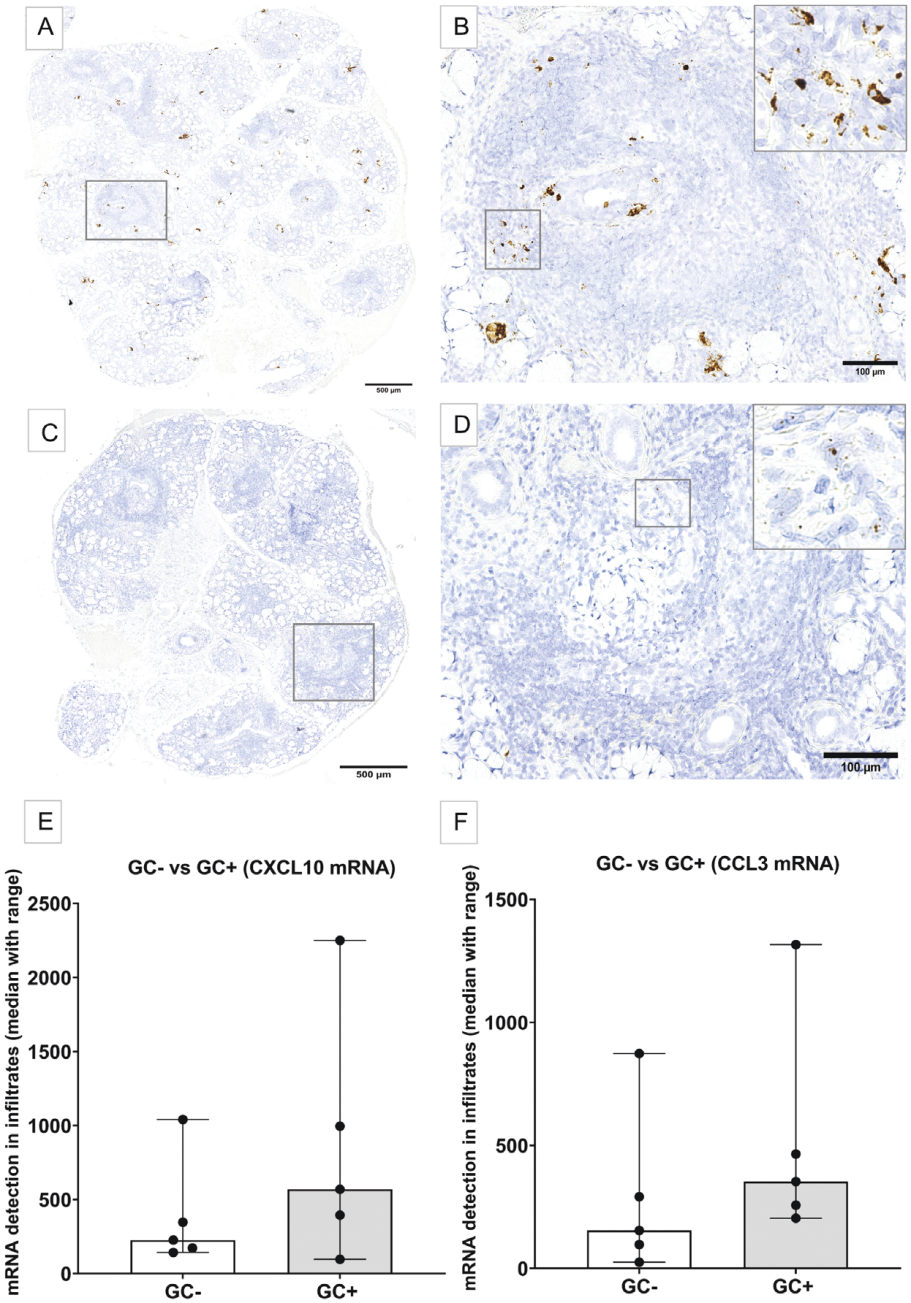
detection of CXCL10 and CCL3 mRNA transcripts within mononuclear cell infiltrates and GC-like formations using RNAscope® *in situ* hybridization. The patient exemplified in A–D has a positive focus score of 8 and displays a GC-like formation. (A) CXCL10 mRNA present within multiple focal infiltrates, where the GC-like formation is evident, displaying a lighter appearing central core, and a darker outer zone, thereby creating an ectopic GC-like formation in the gland. (B) Higher magnification of GC-like structure in (A) representing several mRNA clusters of CXCL10 within the large focal infiltrate, and smaller CXCL10 clusters and single mRNA positive cells around and within the GC. (C) CCL3 mRNA observed within larger infiltrates and GC-like structures as scattered small mRNA molecules, adjacent to normal salivary gland tissue. (D) Higher magnification of square in (C), showing single CCL3 mRNA positive cells. (E) Expression of CXCL10 mRNA detected in pSS patients. Comparison between GC− (white) and GC+ (grey). (F) Expression of CCL3 mRNA comparing GC− pSS patients (white) and GC+ pSS patients (grey)

Both CXCL10 and CCL3 were observed within mononuclear focal infiltrates in the pSS patients, where higher levels of CXCL10 were expressed when compared with CCL3. Interestingly, CXCL10 was mainly localized as clusters and was also detected as single mRNA dots in cells interstitially between normal appearing salivary gland tissue, surrounding both acini and ducts. Meanwhile, CCL3 was seen as scattered single mRNA molecules, expressed predominantly in patients with highest focus scores. CXCL10 and CCL3 mRNA transcripts were mostly detected in the periphery of infiltrates containing GC-like formations. However, some patients also expressed clusters of CXCL10 within GC-like structures. Additionally, patients with a higher focus score had clusters of CXCL10 mRNA and also located in the central zone of GC, while cells expressing single CCL3 mRNA were observed in the inner zone of GC-like formations. Interestingly, CXCL10 and CCL3 mRNA transcripts were not detected in close proximity to adipose tissue areas nor fibrosis (Fig. 2).

Furthermore, quantification of the mRNA expression showed that CXCL10 was generally present in higher levels within the mononuclear infiltrates than CCL3 in pSS patients studied. Also, both CXCL10 and CCL3 mRNA expressions were enriched in GC-positive patients when compared with GC-negative patients but did not show a significant difference (Fig. 2).

### Enhanced CXCL10 mRNA expression in acinar epithelium of pSS salivary glands correlates with prominent clinical features

**Figure 3:**
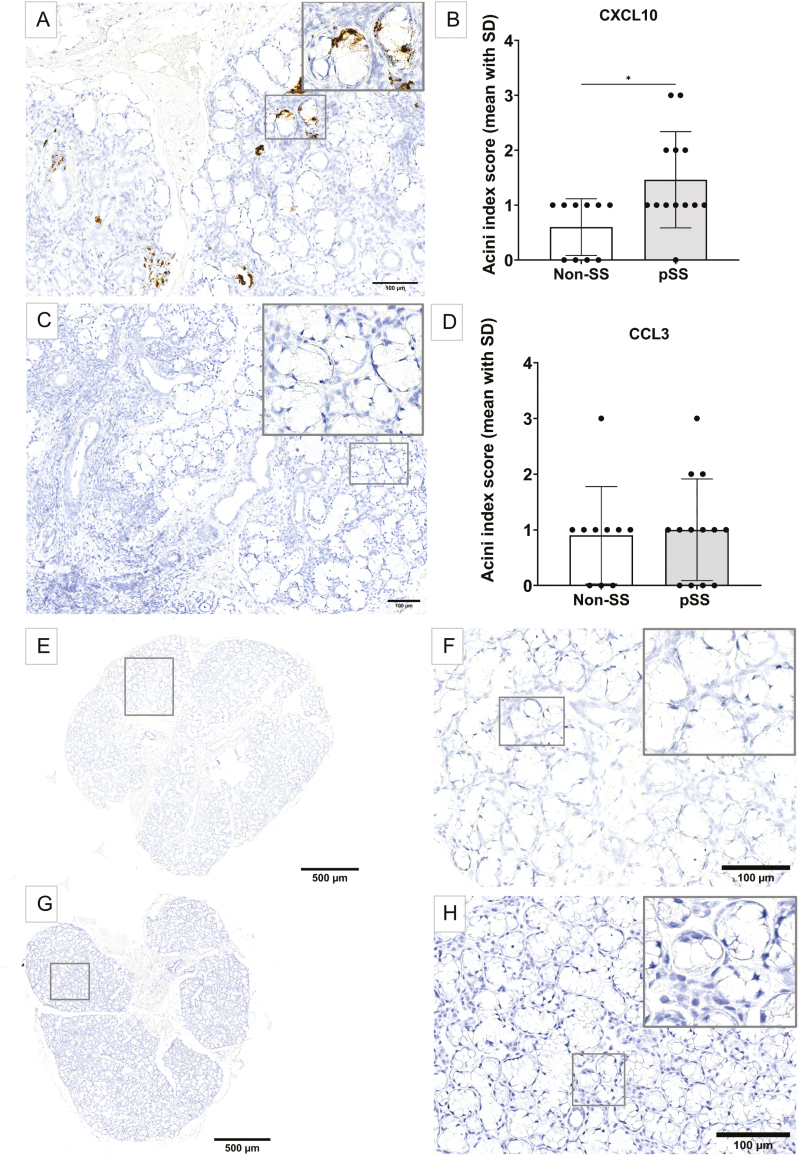
CXCL10 and CCL3 mRNA expression in acinar epithelium of pSS salivary glands compared with non-SS sicca controls. (A) MSG with CXCL10 mRNA clusters (brown) detected in the acinar epithelium of an SSA and/or SSB positive pSS patient. (B) Significantly higher CXCL10 acini index score detected in pSS patients compared with non-SS sicca controls (*P* = 0.019). (C) No clustering of CCL3 mRNA detected in the acini of pSS MSG. (D) Expression of CCL3 acini index score between pSS and non-SS sicca controls showed no significant difference. (E) No CXCL10 mRNA detected in a non-SS sicca subject. (F) Higher magnification of square in (E) in the same non-SS sicca control with negative CXCL10 mRNA detection. (G) No CCL3 mRNA detected in the normal appearing salivary gland of a non-SS sicca control. (H) Higher magnification of square in (G) representing smaller accumulation of immune cells too few to be regarded as a focus score and negative CCL3 mRNA detection

CXCL10 was expressed more evidently in the acini of pSS patients when compared with CCL3. Little to no chemokine expression was observed in the non-SS sicca controls. Moreover, a quantification of CXCL10 mRNA expression in acini (acini index score) revealed significantly enriched CXCL10 transcripts in pSS patients as compared with the non-SS sicca control group. When scoring the glands, the FI and fibrotic areas were excluded and only normal appearing acinar epithelium was analysed. However, the acini index appears not to have been influenced by a higher FI, atrophy, or focal infiltration in pSS MSG for neither CXCL10 nor CCL3 as no mRNA fragments were detected in these tissues (Fig. 3).

**Figure 4:**
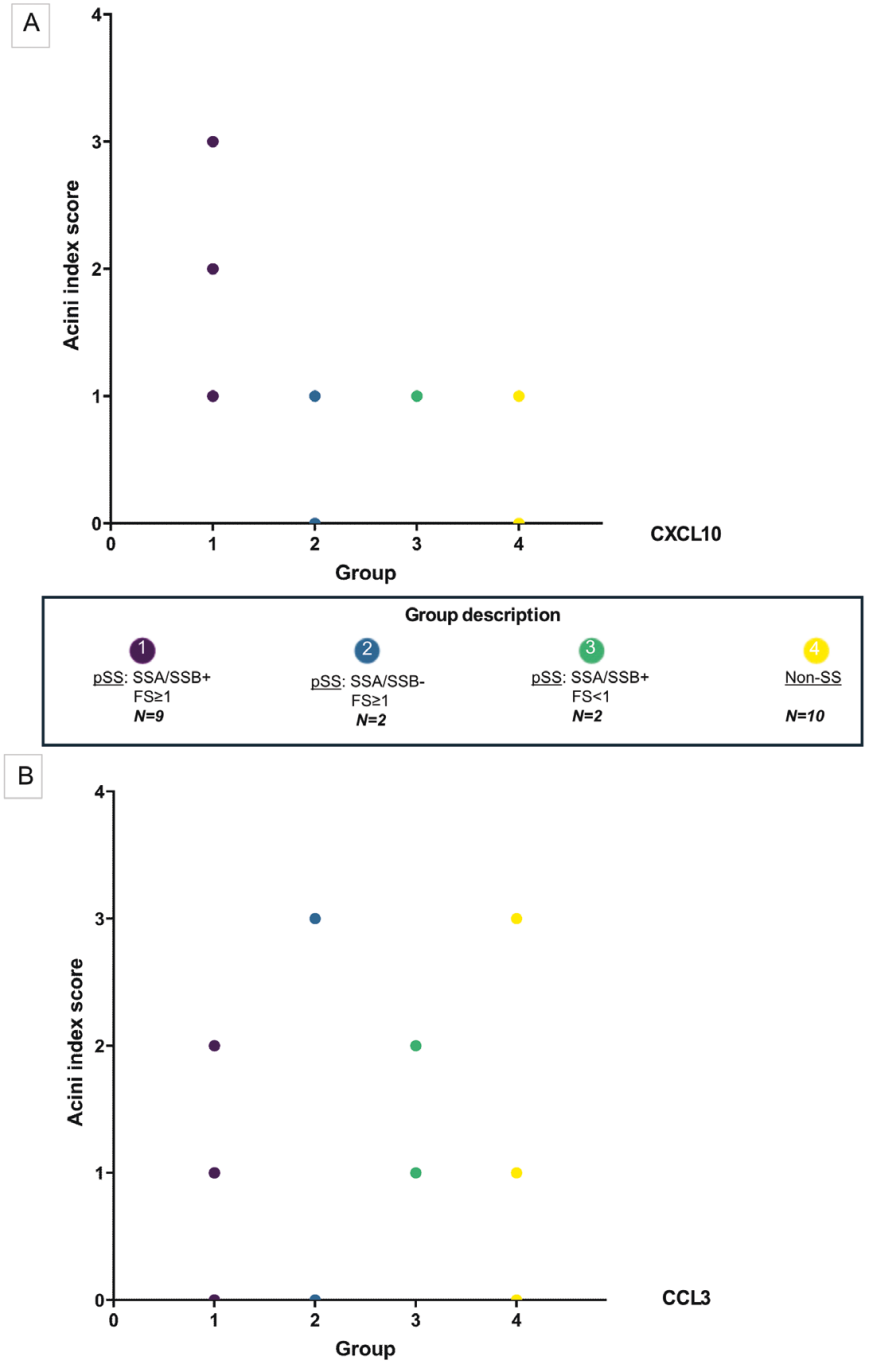
acinar expression of CXCL10 and CCL3 mRNA correlates with disease features of the study population. (A) A bubble plot presenting the acini index score of CXCL10 mRNA (*y*-axis) detected in both pSS and non-SS sicca controls, where 1 represents low, 2 moderate, and 3 prominent levels of the mRNA chemokine observed in the acinar epithelium. (B) A bubble plot presenting the acini index score of CCL3 mRNA detected in both pSS and non-SS sicca controls, estimated as in (A). The colour of the bubbles and *x*-axis represents the different participant subgroups; group 1: SSA and/or SSB positive pSS patients with FS ≥ 1 (*n* = 9), group 2: SSA and SSB negative and FS ≥ 1 pSS patients (*n* = 2), group 3: SSA and/or SSB positive pSS patients with FS < 1 (*n* = 2), and group 4: SSA and SSB negative and FS < 1 non-SS sicca controls (*n* = 10). The *x*-axis represents the patient groups in the following order form pSS in group 1, 2, and 3, and non-SS sicca controls in group 4

Furthermore, the pSS patients were categorized in subgroups based on clinical parameters including SSA and/or SSB autoantibody positivity and focus score to verify whether there was any relation between disease features and mRNA expression levels. The levels of CXCL10 in acinar epithelium seemed to be influenced by the presence of both SSA and/or SSB autoantibodies and focus score. CXCL10 transcripts were most prominent in patients expressing both SSA and SSB autoantibodies and a focus score ≥ 1, and least detected in non-SS sicca controls showing no autoantibody positivity and a negative focus score. On the other hand, CCL3 mRNA transcripts in the acini were more heterogeneously expressed and did not correlate with these disease features in any way (Fig. 4).

### Dual staining for detection of CXCL10 expressing immune cells using immunohistochemistry and RNAscope® *in situ* hybridization

**Figure 5:**
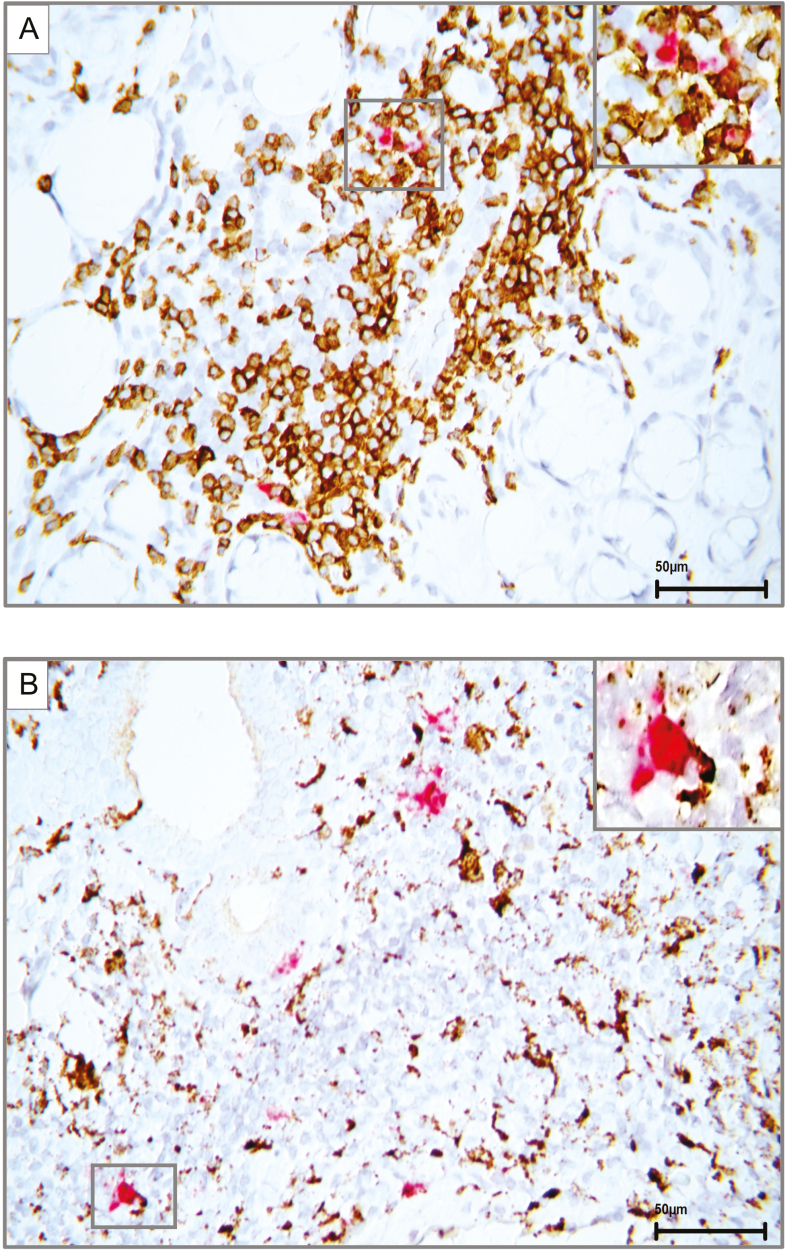
dual staining demonstrating detection of CXCL10 expressing immune cells. (A) Dual staining utilizing *in situ* hybridization in combination with immunohistochemistry in MSG of a pSS patient to detect CXCL10 mRNA (red) expressed alongside CD3 positive T cells (brown), respectively. Higher magnification shows double positive CD3 T cells (brown) expressing clusters of CXCL10 (red). (B) Dual staining employing *in situ* hybridization in combination with immunohistochemistry in MSG of a pSS patient to detect CXCL10 mRNA (red) expressed along with CD68 positive macrophages (brown), respectively. Higher magnification shows a double positive macrophage (brown) also expressing a large cluster of CXCL10 (red)

By utilizing a unique experimental approach where we combined classical immunohistochemical staining with a novel *in situ* hybridization using RNAscope® in MSG of pSS patients, we were able to target the mRNA of the chemokine CXCL10 in combination with CD3 positive T cells and CD68 positive macrophages. This was performed as a dual staining method where the CD3 positive T cells and CD68 positive macrophages were identified through immunohistochemistry and visualized using DAB chromogen (brown), while CXCL10 mRNA expression was detected through *in situ* hybridization and visualized using fast red chromogen. Red clusters of CXCL10 were observed both interstitially and within the focal infiltrates, where CD3-positive T cells and CD68 macrophages also expressed CXCL10 (Fig. 5). CD20-positive B cells were also detected in combination with CXCL10 using the same technique, but the results were not optimal and difficult to quantify. This can be due to the strong enzymatic treatment of the tissue in the dual staining protocol that in the case of anti-CD20, antibody may have weakened the epitopes, and in turn the staining of the B cells (data not shown).

## Discussion

There is a long, withstanding, unmet need for non-invasive diagnostic tools when assessing pSS patients, thus validating the ongoing search for potential disease biomarkers. Since chemokines play a major role in mediating central pathological processes that are associated with autoimmunity, we have previously screened saliva and tear fluid from pSS patients for upregulated cytokines and chemokines that could serve as potential disease biomarkers, and aid in facilitating patient diagnosis [2]. Our results demonstrated significantly upregulated levels of chemokines CXCL10 and CCL3 in both saliva and tear fluid of pSS patients. In this study, we explored the expression of these two specific chemokines in MSG from pSS patients and non-SS sicca controls to gain more insight into their possible contribution in the autoimmune glandular environment within a well-characterized patient cohort. Since such chemokines have previously been difficult to detect through classical immunohistochemistry [20–22], we have applied a systematical quantification and identified the localization of these chemokines using the RNAscope® *in situ* hybridization, to our knowledge, for the first time in glandular MSG tissue from pSS patients (Figs 2 and 3). This allowed the identification of both CXCL10 and CCL3 chemokine mRNA with high specificity and sensitivity [16, 17].

Our results showed CXCL10 as cellular clusters located within focal infiltrates and interstitially in the MSG of pSS patients (Fig. 2A and B). Meanwhile, CCL3 was mainly detected in lower numbers, as single scattered molecules in the periphery of these infiltrates (Fig. 2C and D). Furthermore, the CXCL10 expression levels were directly proportional to increasing focus score. This might be due to the high numbers of CXCR3 expressing CD4 positive T helper cells being a central part of the cellular composition within these focal infiltrates [12].

Interestingly, both chemokines were also observed in close proximity to GC-like structures (Fig. 2E and F), but neither adjacent to fatty tissue nor fibrosis. This could suggest a role for these chemokines in driving the inflammatory process within the glandular tissue in pSS. Our RT-qPCR analysis, although performed on a different set of pSS patients, supports this notion, with a higher chemokine expression level in GC-positive pSS patients, as compared with GC-negative individuals (Fig. 1B). The development of ectopic GC-like structures in the MSG of pSS shares many of the same features as in secondary lymphoid organs, but the mechanisms that trigger this process ectopically remain unclear [23]. Studies on mice have previously shown that chemokines play a central role in lymphocyte recruitment when creating an ectopic GC-like structure, although a particular initial immunological mediator is yet to be defined [23].

To investigate the salivary gland epithelium, an acini index was defined to measure CXCL10 and CCL3 epithelial expression in both pSS patients and non-SS sicca controls (Fig. 3A–D). CXCL10 was more evidently expressed amongst pSS patients, where the highest CXCL10 acini index score was found in patients with prominent disease features including SSA and/or SSB autoantibody positivity and a higher focus score (Fig. 4A). Our RT-qPCR analysis was in line with these observations, showing a significant upregulation of CXCL10 in the analysed material of pSS patients when compared with non-SS sicca controls, as previously reported by Ogawa et al. [10]. These observations suggest that increased expression of the pro-inflammatory CXCL10 chemokine might be a part of the driving force leading to the development of characteristic disease abnormalities within the salivary gland epithelium of pSS patients [24]. However, although our RT-qPCR analysis revealed elevated levels of CXCL10 and CCL3 observed in the MSG of pSS patients supporting our *in situ* hybridization findings (Fig. 1A), these increased levels may also be due to higher numbers of infiltrating cells within the glandular tissue.

Intriguingly, CXCL10 has also been shown to play a central role in the pathogenesis of other autoimmune diseases. For instance, in rheumatoid arthritis [25, 26], similar to pSS, CXCL10 increased the migration of inflammatory cells mediated through CXCR3 binding on CD4-positive T helper cells [27, 28]. Moreover, in multiple sclerosis, CXCL10 is thought to have a pro-inflammatory impact affecting the Th1 subset of CD4-positive T helper cells, thereby influencing disease evolvement [29].

On the other hand, CCL3 glandular acini expression was more heterogeneously displayed among the study participants and did not correlate with disease features (Fig. 4B), although significantly higher levels of CCL3 were detected in our pSS patients through RT-qPCR when compared with the non-SS sicca controls (Fig. 1A). Studies have shown that CCL3 is central in the promotion and migration of inflammatory components to tissues in other autoimmune diseases, particularly rheumatoid arthritis, where it participates in the pathogenesis of cartilage bone destruction [14, 30].

Since lymphocytic infiltration in patients’ salivary glands is a characteristic feature of pSS, we wished to explore the expression pattern of glandular T cells, B cells, and macrophages in combination with CXCL10 at the site of inflammation. Chemokines have previously been difficult to detect and quantify due to their small size and location in the tissue. Although immunohistochemistry has been the golden standard for histological detection of protein in the salivary gland tissue for decades, there are currently no antibodies available to reliably detect and quantify CXCL10 and CCL3 protein in the MSG [20–22, 31]. Consequently, we combined classical immunohistochemical staining with RNAscope® *in situ* hybridization in the MSG of pSS patients. With this, we were able to simultaneously target the mRNA of the chemokine CXCL10 in combination with CD3-positive T cells (comprising of CD4-positive T helper cell subsets and CD8-positive cytotoxic T cells), CD68-positive macrophages, and CD20-positive B cells in the glandular tissue of the patients. Our analysis showed CD3-positive T cells (Fig. 5A) and CD68-positive macrophages also expressing CXCL10 located both interstitially and within the focal infiltrates (Fig. 5B), while our CD20 B cell staining was too weak to be quantified. As aforementioned, this can be due to the strong treatment of the tissue in the dual staining protocol (data not shown). Meanwhile, cells only positive for CXCL10 mRNA were also observed, implying that additional immune cells may also participate in the production of this chemokine within the infiltrates and interstitially.

Nonetheless, our current novel approach has proven to be very precise regarding specificity and sensitivity of mRNA detection [16]. Moreover, by combining RNAscope® for targeting mRNA with other staining techniques such as immunohistochemistry, one can attain a broader understanding of the different cell phenotypes and adjacent chemokines within the infiltrates and normal appearing tissue structures of the salivary gland in pSS [32].

## Conclusions

The application of novel RNAscope® technology in the MSG of pSS patients and non-SS sicca controls allowed for the detection of the chemokines CXCL10 and CCL3 at the site of inflammation. Although both chemokines were present more evidently in pSS patients, CXCL10 expression was most prominent. Moreover, a strong association between the salivary gland expressions of CXCL10 was observed in relation to characteristic disease features in pSS patients, including focal infiltration, GC-like formations, and SSA and/or SSB autoantibody positivity. We therefore suggest CXCL10 as a possible disease biomarker in pSS due to its upregulated expression in both saliva and MSG of pSS patients. The expression pattern of CXCL10 should therefore be re-evaluated in a larger pSS patient cohort, followed by additional functional studies, to further validate its potential as a disease biomarker in pSS.

## Supplementary data

Supplementary data is available at *Clinical and Experimental Immunology* online.

uxae087_suppl_Supplementary_Figures_S1-S2

## Data Availability

All data sets are available upon request.

## Abbreviations:

ACD: advanced cell diagnostics
AOD: assay-on demand TaqMan gene expression assay
CCL3: C-C motif chemokine ligand 3
CXCL10: C-X-C motif chemokine ligand 10
DAB: 3,3'-diaminobenzidine
DAPI: 4',6-diamidino-2-phenylindole
FS: focus score
FI: fatty infiltration
GAPDH: glyceraldehyde 3-phosphate dehydrogenase
GC: germinal centre
HRP: hydrogen peroxide
IFN-γ: interferon gamma
JLJ: Janicke Liaaen Jensen
KS: Kathrine Skarstein
MSG: minor salivary gland
pSS: primary Sjögren’s syndrome
RNA: ribonucleic acid
ROI: region of interest
RT-qPCR: real-time quantitative polymerase chain reaction.
