# Supplementary material for: Increased expression of CXCL10 and CCL3 salivary gland chemokines in primary Sjögren’s syndrome detected and systematically quantified using RNAscope® in situ hybridization
Source: Clin Exp Immunol. 2024 Oct 22;219(1):uxae087. doi: 10.1093/cei/uxae087 (PMC11771196; doi:10.1093/cei/uxae087)
Supplement: uxae087_suppl_Supplementary_Figures_S1-S2 [file uxae087_suppl_Supplementary_Figures_S1-S2.docx]

## **Increased expression of CXCL10 and CCL3 salivary gland chemokines in primary Sjögren’s syndrome detected and systematically quantified using novel RNAscope® *in situ* hybridisation**

**Supplementary Material**

**
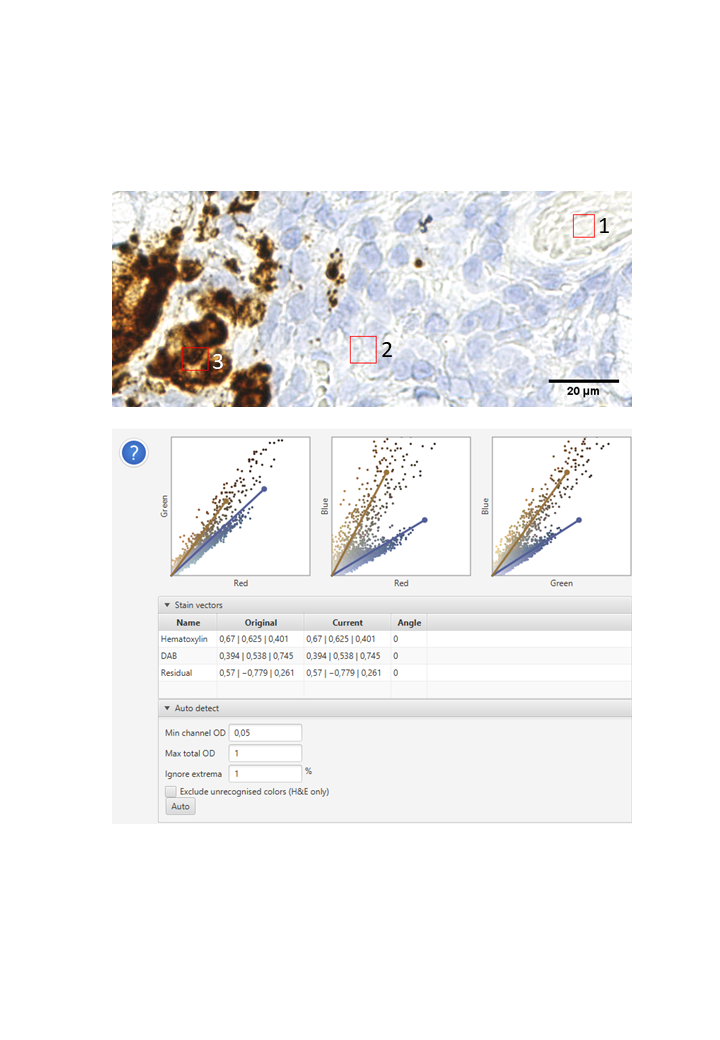
**

**Supplementary Figure 1:** **Visual staining editor used for stain separation in brightfield images.** The scatterplot represents the relationship between the red, blue, and green values for each pixel, and compares the three colours. This was used to separate the haematoxylin, DAB staining and background. A small rectangle was drawn in a representative area of the background (1), haematoxylin (2) and DAB staining (3) to establish these vectors.

###
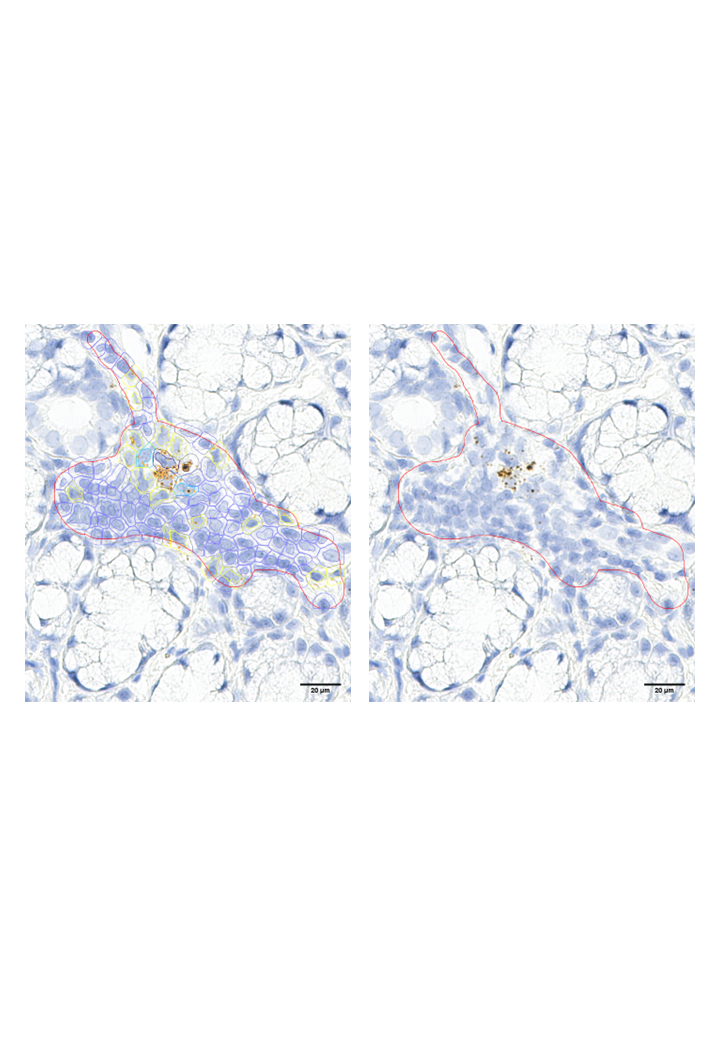


**Supplementary Figure 2:** **Demonstrating analysis of stained focal infiltrate in a pSS patient using the QuPath image software.** The patient exemplified has a positive focus score of 2, and positive SSA and SSB autoantibodies. Brown dots represent the positive mRNA transcripts of CCL3 within the nuclei and/ or cytoplasm of the inflammatory cells within the focal infiltrate. Positive cell detection was carried out in QuPath software using a cell-detection script specified for the haematoxylin, DAB and background staining vectors. The yellow, blue, and black coloured cells represent positive cells, and the purple cells are counted as negative. The small orange fragments detected are the mRNA transcripts, and the colour of the cell varies depending on the numbers of mRNA fragments detected in the cytoplasm and nucleus of the cell.
